# AAindexNC: Estimating the Physicochemical Properties of Non-Canonical Amino Acids, Including Those Derived from the PDB and PDBeChem Databank

**DOI:** 10.3390/ijms252312555

**Published:** 2024-11-22

**Authors:** Yury V. Milchevskiy, Galina I. Kravatskaya, Yury V. Kravatsky

**Affiliations:** 1 Engelhardt Institute of Molecular Biology, Russian Academy of Sciences, Vavilov Str., 32, 119991 Moscow, Russia; gk@eimb.ru (G.I.K.); jiri@eimb.ru (Y.V.K.); 2 Center for Precision Genome Editing and Genetic Technologies for Biomedicine, Engelhardt Institute of Molecular Biology, Russian Academy of Sciences, Vavilov Str., 32, 119991 Moscow, Russia

**Keywords:** amino acids’ physicochemical properties, non-canonical amino acid (ncAA), stepwise regression analysis, Simplified Molecular Input Line Entry System (SMILES), learning models, AAindex, PDB, PDBeChem

## Abstract

The physicochemical properties of amino acid residues from the AAindex database are widely used as predictors in building models for predicting both protein structures and properties. It should be noted, however, that the AAindex database contains data only for the 20 canonical amino acids. Non-canonical amino acids, while less common, are not rare; the Protein Data Bank includes proteins with more than 1000 distinct non-canonical amino acids. In this study, we propose a method to evaluate the physicochemical properties from the AAindex database for non-canonical amino acids and assess the prediction quality. We implemented our method as a bioinformatics tool and estimated the physicochemical properties of non-canonical amino acids from the PDB with the chemical composition presentation using SMILES encoding obtained from the PDBechem databank. The bioinformatics tool and resulting database of the estimated properties are freely available on the author’s website and available for download via GitHub.

## 1. Introduction

The AAindex is a database of numerical indices representing various physicochemical and biochemical properties of amino acids [1]. The AAindex is widely applied across various research fields, including bioinformatics, computational biology, and molecular biology (the three original AAindex manuscripts have been cited more than 2500 times in total). Specifically, it finds uses in the following research activities:

- Studies of protein–protein interactions, by offering the physicochemical properties of amino acids [2,3].
- Evolutionary biology, specifically changes in proteins, especially in understanding how amino acid substitutions can impact protein function over time [4,5,6,7].
- Mutational analysis, by enabling one to understand how point mutations that alter amino acid sequences affect a protein’s properties, stability, or functionality [8,9,10,11,12,13].
- Enzyme studies, by modeling the enzyme activity, stability, and specificity based on the amino acid properties, supporting both experimental and theoretical enzyme research [14,15,16,17].
- Protein structure prediction, by providing numerical values for amino acid properties such as the hydrophobicity, polarity, or molecular weight, which can be crucial in predicting the secondary, local, and tertiary protein structures [18,19,20,21,22,23].
- Drug design and molecular docking, by providing the required values for the binding affinities between the proteins and drug molecules based on amino acid properties (these affinities can be critical to designing molecules that can effectively bind or inhibit specific proteins) [24,25,26,27].
- Protein function annotation, by comparing the amino acid properties with those of known proteins, facilitating classification based on their physicochemical characteristics [28,29,30,31].
- Sequence alignment and homology modeling, by incorporating amino acid substitution matrices into alignment algorithms that reflect the physicochemical differences between amino acids (this can improve the accuracy of sequence homology models that compare proteins according to their functional or structural similarity) [32,33,34,35].
- Machine learning and predictive models in proteomics, via the application of physicochemical properties from the AAindex as features in various machine learning models that predict the protein structure, function, folding patterns, or interaction patterns [21,36,37,38].

In addition to theoretical methods for studying protein–protein and DNA–protein interactions, experimental approaches are also available. Under certain circumstances, these interactions can be measured directly using advanced techniques such as bioluminescence resonance energy transfer (BRET) [39,40] and atomic force microscopy (AFM) [41]. These methods provide the distinct advantage of the direct measurement of specific molecular complexes under study. However, it can be challenging to generalize the data obtained through these methods, and they cannot be explicitly used in theoretical studies or for predicting the structures and properties of proteins and biomolecular complexes. Moreover, these methods cannot be employed for examining compounds that have yet to be synthesized or isolated (for instance, in drug design). Consequently, comprehensive databases of biological macromolecule constituents remain required for many research areas.

The AAindex database was initially posited as a source of physicochemical properties for the 20 canonical amino acids, which are encoded by the triplet codons of the genetic code [1]. Over time, it became clear that proteins include a noticeable amount (more than 1000) of non-canonical amino acids (ncAAs), which play a role in metabolism and take part in signal transduction. Canonical amino acid modifications in vivo are generated through post-translational modifications, backbone alterations (β- and γ-amino acids) [42,43], and stereochemical inversions (D-amino acids) [44]. The number of ncAAs is also significantly increased by chemical syntheses [45]. Many ncAAs inherently resist proteolytic degradation, thus making them potential protease inhibitors and research tools for profiling studies in substrate optimization and enzyme inhibition. ncAAs can also be applied in various fields of research, such as pharmacokinetics and peptidomimetics [46], the thermal stabilization of enzymes [47], enzyme kinetics, protease research [44], molecular interactions, bioimaging, structure–function studies (especially ncAAs with bio-orthogonal labels), and even photo-control to set the protein activity to ON or OFF [48].

Given the broad application of ncAAs in research, accurate physicochemical data for ncAAs are critical across computational molecular modeling tasks. The incorporation of ncAA-specific parameters into traditional force fields through expansion and reparameterization provides the means for the precise modeling of novel side chain interactions, modified backbone conformations, and unique electronic effects [49,50]. These physicochemical properties are also essential for predicting the protein and peptide structure and function, where the hydrophobicity, charge, and size directly affect the folding, stability, and protein complex interactions [51]. Precise ncAA models enhance the structural motif analysis and protein engineering that is essential for stable protein-based drug designs and designing enzymes with novel functions [52]. Furthermore, accurate ncAA physicochemical data improve molecular dynamics simulations via expanded force fields, thereby enhancing the predictions of protein conformations and interaction sites. Machine learning models can also benefit from incorporating these properties [53].

Since ncAAs are, by definition, non-standard, they are absent from most databases. As a result, the design of proteins incorporating ncAAs, functional predictions, and their overall characterization predominantly depend on data from canonical amino acids. The purpose of this study was to develop an algorithm for the accurate prediction of all the physicochemical properties from the AAindex database and to apply it to all known ncAAs, thereby creating an extension of the AAindex database.

## 2. Results

We kindly recommend that readers start with the Methods section to gain a deeper understanding of this methodological manuscript.

We developed a method to evaluate the physicochemical properties from the AAindex database for non-canonical amino acids and assessed its prediction accuracy. The method was implemented as a bioinformatics command-line tool, accessible via the website https://aaindexnc.eimb.ru. Using this tool, we estimated the physicochemical properties of non-canonical amino acids from the PDB, utilizing their chemical compositions represented through SMILES encoding obtained from the PDBechem databank.

As described in Section 4.3, the quality of the predictions was evaluated using the Pearson’s correlation coefficient between the experimental and predicted values for a specific physicochemical property across the 20 canonical amino acids. A learning model was constructed for each of the 566 physicochemical properties from the AAindex database. The quality of the predictions made by each learning model was evaluated statistically in order to assess their accuracy and to estimate the root mean square error of the predicted property.

A command line, cross-platform bioinformatics tool, written in C++, was developed to predict each of the AAindex’s 566 physicochemical properties based on the generated learning models from the SMILES encoding of any ncAA, including those not yet known or synthesized. This tool was applied for each ncAA obtained from the PDB that was present in the PDBeChem databank and did not contain the elements As, B, Br, Cl, F, I, P, or Se (see Section 4.2). The resulting database of the predicted properties is available at https://aaindexnc.eimb.ru (accessed on 17 November 2024) and on GitHub: https://github.com/Milchevskiy/AAindexNC (accessed on 17 November 2024).

We also assessed the prediction quality for each physicochemical property from the AAindex database using the approach described in Section 4.3. The top 20 best predicted physicochemical properties from the AAindex database are presented in Table 1. This table provides insight into the reliability of the models developed for each physicochemical property. A higher correlation coefficient obtained during model optimization (in the leave-one-out mode) indicates greater reliability in predicting the given property for ncAAs. Additionally, the root mean square error (RMSE) for the selected property is presented, along with the number of significant predictors included in the model and the Fisher statistic threshold at which the model achieved the maximum correlation coefficient.

The complete version of Table 1, which encompasses all 566 physicochemical properties from the AAindex database, is presented in Supplemental Table S1.

A moderate level of correlation (*r*_j-n_ > 0.515, *p*-value < 0.01) was achieved for the prediction of 227 properties, while an average level of correlation (*r*_j-n_ > 0.378, *p*-value < 0.05) was obtained for 322 properties. The method we developed performs significantly better in predicting the physicochemical properties that are measured via experiments and less effectively for those that can be obtained through straightforward computation. Notably, 72.7% of the hydrophobicity-related properties were predicted with *r*_j-n_ > 0.5 (*p* < 0.012), and 93.9% of these properties were predicted with at least an average level of correlation *r*_j-n_ > 0.37 (mean *r*_j-n_ for all hydrophobicity values = 0.621, mean RMSE for all hydrophobicity values = 0.239) (Table 2).

In order to ascertain which components of an amino acid contribute to a particular physicochemical property, it is possible to analyze which predictors were found to be statistically significant for predicting that property. In the case of the EISD840101 (consensus normalized hydrophobicity scale) [54] property, the most statistically significant predictors were the number of oxygen atoms, the number of positive charges, the number of nitrogen atoms, and the number of carbon atoms in aromatic rings. The learning model for the EISD840101 property is presented in Table 3.

The identification of amino acid components that exert the greatest influence on a given physicochemical property allows for the design, creation, or modification of ncAAs with enhanced or reduced desired properties (e.g., hydrophobicity) through chemical synthesis. Information regarding the significance of specific predictors is typically unavailable in predictions made by neural networks.

Let us consider, e.g., how the prediction of the EISD840101 property value for the HYP (4-HYDROXYPROLINE) ncAA is performed. OpenEye’s SMILES for HYP can be recorded as O[C@H]1CN[C@@H](C1)C(O)=O, and the predictors are represented as shown in Table 4, which contains the predictor values corresponding to this SMILES, along with the corresponding regression coefficients and the constant term.

Omitting the zero terms, EISD840101 (HYP) = (−0.563688) × 3 + (−0.556289) × 1 + 0.11307 × 5 + (−0.290756) × 1 + 1.629829 = −0.34293. The RMSE value for this property, calculated for the 20 canonical amino acids, is 0.282 (Supplemental Table S1). When predicting the physicochemical properties of ncAAs, we utilized the RMSE (the most commonly applied error function) to fit the prediction model. So, the predicted value for EISD840101 (HYP) is −0.343 ± 0.282. Similarly, all the physicochemical properties from the AAindex database were calculated for all ncAAs.

Thus, our database comprises a series of correlation coefficients (which can be used as a measure of the prediction quality), regression coefficients, their associated statistical significance (*F*-values), RMSE values, and a comprehensive list of predictors forming the model for each physicochemical property in the AAindex database. This dataset may be downloaded for local analysis or accessed via a web browser.

## 3. Discussion

In this study, we introduced a method for evaluating the physicochemical properties listed in the AAindex database.

Some properties, such as FASG760101 (molecular weight) [55] or CHAM830106 (the number of bonds in the longest chain) [56], can be calculated straightforwardly for any molecule based on its chemical composition. The AAindex database also contains many properties derived from the amino acid occurrence analysis (e.g., DAYM780101, the amino acid composition [57]) or the conformational preferences of amino acids (e.g., CHOP780101, the normalized frequency of beta-turns [58]). Predicting such physicochemical properties is unnecessary and of little interest, since they can be computed straightforwardly.

In contrast, the situation is entirely different for the physicochemical properties obtained from experiments. The AAindex database includes a wide variety of scales for hydrophobicity, the energy of transfer between mediums, polarizability, isoelectric points, solvation-related properties, etc. The accurate prediction of these properties can significantly enhance the learning models for proteins containing ncAAs, particularly when these models employ amino acid physicochemical properties from the AAindex database as predictors [18,21].

An example of the necessity for using physicochemical properties for ncAAs can be seen in our previous work [21], where we encountered challenges in constructing an adequate learning model for protein secondary and local structure prediction, using the physicochemical properties from the AAindex databases (specifically, hydrophobicity) as predictors. The prediction accuracy was significantly lower for collagen and for globular proteins containing collagen-like regions. This may be due to the high proportion of the ncAA hydroxyproline in collagen and collagen-like regions, as it seems that hydroxyproline plays a key role in stabilizing the structure of collagen [59]. Moreover, a quantitative relationship has been identified between collagen melting temperatures across various species and the percentage of hydroxyproline residues [60], supporting hydroxyproline’s role in stabilizing collagen-like structures, as structural stability is closely tied to the melting temperature. Despite recent advances, the accurate prediction of collagen and collagen-like conformations remains challenging [61]. We propose that one reason for these difficulties is the lack of distinction between proline and hydroxyproline in both protein sequence and physicochemical property databases, e.g., the abovementioned property EISD840101 (consensus normalized hydrophobicity scale) reveals a fivefold difference between measured values for proline and predicted values for hydroxyproline (−0.07 for proline and −0.34 for hydroxyproline, with *r*_j-n_ = 0.924). An even more pronounced distinction appears for the ROSM880101 (side chain hydropathy, uncorrected for solvation) [62] property, where the value for proline is −1.75, while the predicted value for hydroxyproline is 2.96, with *r*_j-n_ = 0.973.

We plan to refine learning models for both local and secondary protein structure prediction by incorporating the predicted physicochemical properties of hydroxyproline and other ncAAs (using predictions with *r*_j-n_ ≥ 0.6) in our future research.

## 4. Materials and Methods

### 4.1. Formulation of the Problem

In order to formalize the problem of predicting the physicochemical properties from the AAindex database for ncAAs, it is necessary to include information on the components shared between canonical and non-canonical amino acids in the prediction model. In particular, for each canonical amino acid, a feature set corresponding to its chemical composition must be generated. Subsequently, this set can then be correlated with a physicochemical property from the AAindex database. This defines the problem, which we solved using stepwise regression analysis.

As a feature set, we applied a set of predictors, derived from the SMILES (Simplified Molecular Input Line Entry) encoding [63,64] for each amino acid. SMILES is a string notation that is used to describe the structure of chemical compounds using short sequences [63,64,65]. These SMILES strings can be conveniently imported by the majority of computer molecule editors and converted back into two-dimensional diagrams or three-dimensional molecular models. SMILES encoding is also a widely utilized method for generating features in problems related to the prediction of chemical structure and function. This includes applications with RDKit [66], Dragon [67], CDK2 [68], PyDescriptor [69], and others. The substitution of chemical compounds with their components is successfully applied in machine learning, including the use of SMILES encoding [70,71,72,73]. SMILES can be utilized for classification on the basis of images of chemical compounds [74].

Among the non-canonical amino acids found in the PDB [75], some contain chemical elements not present in the 20 canonical amino acids, for which the AAindex database was created. It was necessary to exclude these amino acids from further consideration.

### 4.2. SMILES for Canonical Amino Acids

The learning model was constructed using all the canonical amino acids. Based on the SMILES encoding for these amino acids, we selected the features that described each amino acid in the most accurate way (using the statistical assessment described below), which were then mapped to the values of each of the 566 properties from the AAindex database. There are multiple standards for SMILES notation. Given our focus on isomeric properties, particularly the presence of chiral centers, we utilized the most recent version of the SMILES standard that incorporates these features (canonical SMILES calculated using the OpenEye OEToolkit version 1.5.0). The SMILES for all the canonical amino acids were obtained from the PDBeChem server [76] via the following URL: https://www.ebi.ac.uk/pdbe-srv/pdbechem/chemicalCompound/show/XXX (accessed on 17 November 2024), where XXX represents the amino acid PDB code (e.g., LEU, ALA, etc.). The SMILES for all the canonical amino acids can be found in Supplemental Table S2.

### 4.3. SMILES for Non-Canonical Amino Acids

The complete (to September of 2024) Protein Data Bank, PDB [75], was downloaded from ftp://rsync.wwpdb.org (accessed on 17 November 2024) using the rsyncPDB script, available at https://files.wwpdb.org/pub/pdb/software/rsyncPDB.sh (accessed on 10 September 2024). The downloaded PDB was analyzed using an ad hoc Python v.3.10 script that utilized the ProDy v.2.4.1 [77] library for the purpose of conducting an analysis of the Polymer.modified list for each protein. This list comprises all the non-canonical/modified amino acids of the protein, if they are present. The total number of ncAAs present in the PDB is considerable: a comprehensive analysis revealed that all the PDB proteins contained 167,477 non-unique ncAAs, with 1162 unique ncAAs. From this primary set, all the non-canonical amino acids (ncAAs) containing chemical elements not incorporated in classical amino acids (As, B, Br, Cl, F, I, P, Se) were excluded, as our approach is unable to predict the influence of these elements on the physicochemical properties of the appropriate ncAAs. The SMILES for ncAAs was obtained from the PDBeChem server [76] using an ad hoc Python v.3.10 script that utilized the server’s JSON API, accessible via the following URL: https://www.ebi.ac.uk/pdbe/api/pdb/compound/summary/XXX (accessed on 17 November 2024), where XXX is the code of the required chemical compound. All scripts are available on GitHub: https://github.com/Milchevskiy/AAindexNC (accessed on 17 November 2024).

Table 5 lists the 25 most frequently occurring (more than 70% of the total) ncAAs predicted using our method.

The complete occurrence table for all non-canonical amino acids can be found in Supplemental Table S3.

We selected the SMILES components shown in Table 6 to create the features of the prediction model.

The selection of the optimal set for generating predictors was conducted by testing various sets of predictors (including amino acid component frequencies, amino acid component occurrence polynomial functions, reverse component frequencies, etc.). As a result, the best learning model in terms of the overall performance was achieved using the predictors representing the frequency of the components listed in Table 2, e.g., for alanine (ALA), the predictor corresponding to component ‘C’ is 2, while the predictor corresponding to the component ‘S’ is 0.

In this way, we created a learning model where the dependent variable was a physicochemical property from the AAindex database, and the feature set was the same across all amino acids.

Stepwise regression analysis was used to create learning models describing the relationship between the input feature set and each of the 566 properties in the AAindex database. The physicochemical property FAUJ880111 (positive charge) [78] exhibited a perfect correlation with the predictor, which represents the number of positive charges. Consequently, the calculation of the statistical properties, such as the standard deviation of the regression coefficients, is not feasible and has no meaning. In the case of a perfect correlation, the standard deviation equals zero, and by definition, the Fisher statistic is calculated as the square of the ratio between the regression coefficient and the standard deviation, making the statistic undefined.

Due to the small sample size, comprising only 20 canonical amino acids, we were unable to apply neural networks to predict the physicochemical properties of ncAAs, as the neural networks trained on such a small dataset would suffer from overfitting [79]. Statistical assessments were performed to identify the significant predictors for each amino acid–physicochemical property relationship. These learning models provided templates for the prediction of the ncAAs’ properties.

### 4.4. The Selection of Statistically Significant Predictors and Prediction Quality Statistical Assessment

The final training dataset was relatively small, comprising only 20 standard amino acids, while the number of predictors (13) was comparable to the number of amino acids. Learning models created under such conditions can recognize the existing data satisfactorily but perform moderately when predicting unknown data. To assess the prediction quality, we generated auxiliary models where 1 amino acid was removed from the dataset: based on the subset of 19 canonical amino acids, the given physicochemical property of the 20th amino acid was predicted. The selection of significant predictors for prediction was determined by the value of the Fisher statistic: the predictors were considered significant if, when included in the learning model, the Fisher statistic value for each predictor within the model exceeded the current threshold. By performing this procedure for all 20 canonical amino acids, we obtained the predicted values for the given physicochemical property and calculated the correlation coefficient between the predicted and actual values. The threshold for the F-statistic (*F*-value) was determined via leave-one-out cross validation. The choice of the optimal *F*-value for the physicochemical property EISD840101 (consensus normalized hydrophobicity scale) [54] is shown in Table 7.

This procedure was repeated for all the physicochemical properties from the AAindex database.

## 5. Conclusions

We suggest that the obtained results will be of significant interest for the detailed prediction and analysis of the structure and function of both native and synthetic proteins containing ncAAs. Furthermore, the AAindex physicochemical properties are especially valuable for studies involving small proteins and protein ligands that incorporate ncAAs.

The method that we developed is general and limited only by the absence of experimental data for ncAAs containing chemical elements that are not incorporated in canonical amino acids (As, B, Br, Cl, F, I, P, and Se, at the time of publication). As experimental data for these physicochemical properties are obtained, the learning models can be retrained to include ncAAs with these chemical elements, and the set of statistically significant predictors will be appropriately expanded, so the database can be expanded. We plan to update both the database and learning models as such experimental data become available in the future.

The developed tools (including the source code) and the database are freely accessible at https://aaindexnc.eimb.ru (accessed on 17 November 2024) and on GitHub at https://github.com/Milchevskiy/AAindexNC (accessed on 17 November 2024). These tools can be applied for predicting the individual physicochemical properties of unknown ncAAs, as well as in bioinformatics pipelines for large-scale screening.

The relevance and importance of our work is derived from the increasing acknowledgement of the potential of non-canonical amino acids in enzymology, biocatalysis, and biological therapeutics. We anticipate that our results and the software we developed will facilitate useful theoretical predictions, thereby serving as a foundation for screening studies in these fields.

## Figures and Tables

**Table 1:** The top 20 best predicted physicochemical properties from the AAindex database.

AAindex Accession	*r*_j-n_	RMSE	*F*-Value of *r*_j-n_	*P*_num_
CHAM820101	0.999	0.005	1.2	10
KARS160117	0.994	1.820	2.0	8
FAUJ880103	0.989	0.287	1.1	10
LEVM760105	0.989	0.070	2.1	6
BIGC670101	0.986	4.580	1.0	9
GOLD730102	0.985	6.860	6.9	5
KARS160107	0.982	0.748	4.0	6
CHOC750101	0.976	9.540	1.5	9
FASG760101	0.975	6.920	1.4	10
KARS160101	0.975	0.526	3.4	6
ROSM880101	0.973	1.230	1.7	9
TSAJ990101	0.970	9.920	2.3	7
LEVM760102	0.966	0.255	3.3	6
TSAJ990102	0.966	10.600	3.2	7
KRIW790103	0.963	9.900	4.6	5
KUHL950101	0.958	0.095	3.3	3
ZIMJ680104	0.958	0.850	1.0	7
CHOC760101	0.957	12.500	1.0	7
KARS160114	0.954	2.210	1.5	6
KARS160102	0.952	0.740	3.4	4

The complete list of physicochemical properties from the AAindex database is available at https://www.genome.jp/aaindex/AAindex/list_of_indices (accessed on 17 November 2024); the detailed description of each physicochemical property can be accessed at https://www.genome.jp/entry/aaindex:XXXXX (accessed on 17 November 2024), where XXXXX is the property’s accession code listed in the first column; *r*_j-n_: Pearson’s correlation coefficient between the experimental and predicted values calculated using the leave-one-out cross validation approach; RMSE: root mean square error. The RMSE value depends upon the absolute value of the specific AAindex property. *F*-value of *r*_j-n_: F-statistic value, corresponding to the maximum of *r*_j-n_; *P*_num_: the number of statistically significant predictors for which the *F*-value is above the threshold in the third column.

**Table 2:** Prediction quality of hydrophobicity-related physicochemical properties.

AAindex Accession	*r*_j-n_	Property Explanation
KUHL950101	0.958	Hydrophilicity scale
WOLR790101	0.954	Hydrophobicity index
EISD840101	0.924	Consensus normalized hydrophobicity scale
KIDA850101	0.903	Hydrophobicity-related index
PRAM900101	0.888	Hydrophobicity
ENGD860101	0.887	Hydrophobicity index
BLAS910101	0.873	Scaled side chain hydrophobicity values
GOLD730101	0.828	Hydrophobicity factor
COWR900101	0.808	Hydrophobicity index, 3.0 pH
CIDH920102	0.802	Normalized hydrophobicity scales for beta-proteins
JURD980101	0.774	Modified Kyte–Doolittle hydrophobicity scale
WILM950101	0.714	Hydrophobicity coefficient in RP-HPLC, C18 with 0.1%TFA/MeCN/H_2_O
CIDH920105	0.697	Normalized average hydrophobicity scales
ARGP820101	0.659	Hydrophobicity index
JOND750101	0.646	Hydrophobicity
CIDH920104	0.625	Normalized hydrophobicity scales for alpha-/beta-proteins
CASG920101	0.600	Hydrophobicity scale from native protein structures
PONP800106	0.589	Surrounding hydrophobicity in turn
CIDH920103	0.563	Normalized hydrophobicity scales for alpha-proteins
SWER830101	0.563	Optimal matching hydrophobicity
PONP800103	0.545	Average gain ratio in surrounding hydrophobicity
CIDH920101	0.537	Normalized hydrophobicity scales for alpha-proteins
MANP780101	0.512	Average surrounding hydrophobicity
PONP930101	0.501	Hydrophobicity scales
FASG890101	0.479	Hydrophobicity index
PONP800105	0.476	Surrounding hydrophobicity in beta-sheet
PONP800102	0.458	Average gain in surrounding hydrophobicity
ZIMJ680101	0.455	Hydrophobicity
PONP800101	0.445	Surrounding hydrophobicity in folded form
WILM950102	0.427	Hydrophobicity coefficient in RP-HPLC, C8 with 0.1%TFA/MeCN/H_2_O
WILM950103	0.368	Hydrophobicity coefficient in RP-HPLC, C4 with 0.1%TFA/MeCN/H_2_O
PONP800104	0.242	Surrounding hydrophobicity in alpha-helix
WILM950104	−0.193	Hydrophobicity coefficient in RP-HPLC, C18 with 0.1%TFA/2-PrOH/MeCN/H_2_O

The complete list of physicochemical properties from the AAindex database is available at https://www.genome.jp/aaindex/AAindex/list_of_indices (accessed on 17 November 2024); the detailed description of each physicochemical property can be accessed at https://www.genome.jp/entry/aaindex:XXXXX (accessed on 17 November 2024), where XXXXX is the property’s accession code listed in the first column; *r*_j-n_: Pearson’s correlation coefficient between the experimental and predicted values calculated using the leave-one-out cross validation approach.

**Table 3:** Learning model of AAindex property EISD840101.

*F*-Value	Regression Coefficient	SD of RC	Predictor
507.614	−0.563688	0.025	O
120.263	−0.556289	0.050	N
9.014	0.163592	0.054	n
11.118	0.452579	0.135	N=
56.734	0.113070	0.015	C
111.808	0.148830	0.014	c
22.228	−0.248443	0.052	S
68.744	−0.290756	0.035	c1
44.738	−0.688098	0.102	c2
189.686	−1.168294	0.084	+
Constant term			1.630

*F*-value: the value of the F-statistic used as the threshold for a predictor to be included in the learning model; regression coefficient values were obtained through the stepwise regression procedure; SD of RC: the standard deviation of the regression coefficient across the 20 canonical amino acids.

**Table 4:** The calculation of the EISD840101 property value for the 4-HYDROXYPROLINE ncAA.

Component	Predictor’s Value	Regression Coefficient
=O	1	0.000000
O	3	−0.563688
N	1	−0.556289
n	0	0.163592
=N	0	0.452579
C	5	0.113070
c	0	0.148830
[C@	2	0.000000
S	0	−0.248443
C1	1	−0.290756
C2	0	−0.688098
=	1	0.000000
+	0	−1.168294
Constant term		1.629829

**Table 5:** The 25 most frequently occurring ncAAs in the PDB, predicted using the suggested method.

Code	Amino Acid	SMILES	Number	Percent
MLY	N-DIMETHYL-LYSINE	CN(C)CCCC[C@H](N)C(O)=O	5324	20.151
HYP	4-HYDROXYPROLINE	O[C@H]1CN[C@@H](C1)C(O)=O	2264	8.569
NAG	2-ACETAMIDO-2-DEOXY-BETA-D-GLUCOPYRANOSE	CC(=O)N[C@H]1[C@H](O)O[C@H](CO)[C@@H](O)[C@@H]1O	983	3.721
CSO	S-HYDROXYCYSTEINE	N[C@@H](CSO)C(O)=O	890	3.369
CRO	{2-[(1R,2R)-1-AMINO-2-HYDROXYPROPYL]-4-(4-HYDROXYBENZYLIDENE)-5-OXO-4,5-DIHYDRO-1H-IMIDAZOL-1-YL}ACETIC ACID	C[C@@H](O)[C@H](N)C1=N\C(=C/c2ccc(O)cc2)C(=O)N1CC(O)=O	886	3.354
KCX	LYSINE NZ-CARBOXYLIC ACID	N[C@@H](CCCCNC(O)=O)C(O)=O	877	3.319
PCA	PYROGLUTAMIC ACID	OC(=O)[C@@H]1CCC(=O)N1	783	2.964
CME	S,S-(2-HYDROXYETHYL)THIOCYSTEINE	N[C@@H](CSSCCO)C(O)=O	679	2.570
CSD	3-SULFINOALANINE	N[C@@H](C[S](O)=O)C(O)=O	617	2.335
NRQ	{(4Z)-4-(4-HYDROXYBENZYLIDENE)-2-[3-(METHYLTHIO)PROPANIMIDOYL]-5-OXO-4,5-DIHYDRO-1H-IMIDAZOL-1-YL}ACETIC ACID	CSCCC(=N)C1=N\C(=C/c2ccc(O)cc2)C(=O)N1CC(O)=O	604	2.286
CR2	{(4Z)-2-(AMINOMETHYL)-4-[(4-HYDROXYPHENYL)METHYLIDENE]-5-OXO-4,5-DIHYDRO-1H-IMIDAZOL-1-YL}ACETIC ACID	NCC1=N\C(=C/c2ccc(O)cc2)C(=O)N1CC(O)=O	521	1.972
CGU	GAMMA-CARBOXY-GLUTAMIC ACID	N[C@@H](CC(C(O)=O)C(O)=O)C(O)=O	507	1.919
GYC	[(4Z)-2-[(1R)-1-AMINO-2-MERCAPTOETHYL]-4-(4-HYDROXYBENZYLIDENE)-5-OXO-4,5-DIHYDRO-1H-IMIDAZOL-1-YL]ACETIC ACID	N[C@@H](CS)C1=N\C(=C/c2ccc(O)cc2)C(=O)N1CC(O)=O	396	1.499
CRQ	[2-(3-CARBAMOYL-1-IMINO-PROPYL)-4-(4-HYDROXY-BENZYLIDENE)-5-OXO-4,5-DIHYDRO-IMIDAZOL-1-YL]-ACETIC ACID	NC(=O)CCC(=N)C1=N\C(=C/c2ccc(O)cc2)C(=O)N1CC(O)=O	381	1.442
MDO	{2-[(1S)-1-AMINOETHYL]-4-METHYLIDENE-5-OXO-4,5-DIHYDRO-1H-IMIDAZOL-1-YL}ACETIC ACID	C[C@H](N)C1=NC(=C)C(=O)N1CC(O)=O	356	1.347
OCS	CYSTEINESULFONIC ACID	N[C@@H](C[S](O)(=O)=O)C(O)=O	352	1.332
FME	N-FORMYLMETHIONINE	CSCC[C@H](NC=O)C(O)=O	300	1.136
ORN	L-ORNITHINE	NCCC[C@H](N)C(O)=O	299	1.132
ABA	ALPHA-AMINOBUTYRIC ACID	CC[C@H](N)C(O)=O	284	1.075
CR8	2-[1-AMINO-2-(1H-IMIDAZOL-5-YL)ETHYL]-1-(CARBOXYMETHYL)-4-[(4-OXOCYCLOHEXA-2,5-DIEN-1-YLIDENE)METHYL]-1H-IMIDAZOL-5-OLATE	N[C@@H](Cc1[nH]cnc1)c2nc(C=C3C=CC(=O)C=C3)c([O-])n2CC(O)=O	279	1.056
TYS	O-SULFO-L-TYROSINE	N[C@@H](Cc1ccc(O[S](O)(=O)=O)cc1)C(O)=O	276	1.045
SMC	S-METHYLCYSTEINE	CSC[C@H](N)C(O)=O	258	0.977
M3L	N-TRIMETHYLLYSINE	C[N+](C)(C)CCCC[C@H](N)C(O)=O	254	0.961
ALY	N(6)-ACETYLLYSINE	CC(=O)NCCCC[C@H](N)C(O)=O	239	0.905
GYS	[(4Z)-2-(1-AMINO-2-HYDROXYETHYL)-4-(4-HYDROXYBENZYLIDENE)-5-OXO-4,5-DIHYDRO-1H-IMIDAZOL-1-YL]ACETIC ACID	N[C@@H](CO)C1=N\C(=C/c2ccc(O)cc2)C(=O)N1CC(O)=O	225	0.852

The image and a detailed description of each ncAA can be found in the PDBeChem databank at https://www.ebi.ac.uk/pdbe-srv/pdbechem/chemicalCompound/show/XXX (accessed 17 November 2024), where XXX is the compound code of the ncAA listed in the first column.

**Table 6:** SMILES components selected to create features.

Component	Description
=O O=	oxygen, forming a double bond
O	any oxygen
N	nitrogen, except an aromatic ring
n	nitrogen in an aromatic ring
=N N= =[N	nitrogen, forming a double bond
C	carbon, except an aromatic ring
c	carbon in an aromatic ring
[C@	carbon as a chiral center
S	sulfur
c1 C1 n1 N1 S1	any ring (aromatic or any other cycle)
c2 C2 n2 N2 S2	second ring (aromatic or any other cycle)
=	any double bond
+	positive charge

**Table 7:** The selection of the optimal F-statistic value for the EISD840101 AAindex property.

*F*-Value = 1			*F*-Value = 2			*F*-Value = 2.4			*F*-Value = 3		
***r***	***r*_j-n_**	***P*_num_**	***r***	***r*_j-n_**	***P*_num_**	***r***	***r*_j-n_**	***P*_num_**	***r***	***r*_j-n_**	***P*_num_**
0.997	0.850	11	0.997	0.848	10	0.953	**0.924**	10	0.953	0.890	3

*r*: Pearson’s correlation coefficient between the experimental and predicted values for all 20 canonical amino acids included in the learning model; *r*_j-n_: Pearson’s correlation calculated using the leave-one-out cross validation approach; *P*_num_: the number of statistically significant predictors included in the learning model. No other *F*-values produced higher or equivalent *r*_j-n_ values. Thus, the highest correlation coefficient, *r*_j-n_ = 0.924, corresponds to *F*-value = 2.4 and to the 10 relevant statistically significant predictors used to predict the EISD840101 AAindex property.

## Data Availability

The source code for all scripts used in this work, the final learning model, and the precomputed database of the physicochemical properties of non-canonical amino acids are available at https://aaindexnc.eimb.ru (accessed on 17 November 2024) and on Github at https://github.com/Milchevskiy/AAindexNC (accessed on 17 November 2024).

## Supplementary Materials

The supporting information can be downloaded at https://www.mdpi.com/article/10.3390/ijms252312555/s1.
