# Supplementary material for: AAindexNC: Estimating the Physicochemical Properties of Non-Canonical Amino Acids, Including Those Derived from the PDB and PDBeChem Databank
Source: Int J Mol Sci. 2024 Nov 22;25(23):12555. doi: 10.3390/ijms252312555 (PMC11641631; doi:10.3390/ijms252312555)
Supplement: Supplementary file 1 [file ijms-25-12555-s001.zip › Table S2.pdf]

Supplemental Table S2. Representation of 20 canonical amino acids by SMILES encoding. These so called "Canonical SMILES" were calculated by OpenEye OEToolkit version 1.5.0 and downloaded from PDBeChem server via the following URL: http://www.ebi.ac.uk/PDBeChem/chemicalCompound/show/XXX, where XXX represents the amino acid PDB code (e.g., LEU, ALA, etc.)

Three-letter code	One-letter code	Amino acid OpenSMILES reprepresentation
ALA	A	C[C@@H](C(=O)O)N
CYS	C	C([C@@H](C(=O)O)N)S
ASP	D	C([C@@H](C(=O)O)N)C(=O)O
GLU	E	C(CC(=O)O)[C@@H](C(=O)O)N
PHE	F	c1ccc(cc1)C[C@@H](C(=O)O)N
GLY	G	C(C(=O)O)N
HIS	H	c1c([nH+]c[nH]1)C[C@@H](C(=O)O)N
ILE	I	CC[C@H](C)[C@@H](C(=O)O)N
LYS	K	C(CC[NH3+])C[C@@H](C(=O)O)N
LEU	L	CC(C)C[C@@H](C(=O)O)N
MET	M	CSCC[C@@H](C(=O)O)N
ASN	N	C([C@@H](C(=O)O)N)C(=O)N
PRO	P	C1C[C@H](NC1)C(=O)O
GLN	Q	C(CC(=O)N)[C@@H](C(=O)O)N
ARG	R	C(C[C@@H](C(=O)O)N)CNC(=[NH2+])N
SER	S	C([C@@H](C(=O)O)N)O
THR	T	C[C@H]([C@@H](C(=O)O)N)O
VAL	V	CC(C)[C@@H](C(=O)O)N
TRP	W	c1ccc2c(c1)c(c[nH]2)C[C@@H](C(=O)O)N
TYR	Y	c1cc(ccc1C[C@@H](C(=O)O)N)O
